# Molecular phylogeny and distribution of dengue virus serotypes circulating in Nepal in 2017

**DOI:** 10.1371/journal.pone.0234929

**Published:** 2020-07-07

**Authors:** Sabita Prajapati, Rajindra Napit, Anup Bastola, Ramanuj Rauniyar, Srijan Shrestha, Mahesh Lamsal, Anurag Adhikari, Parmananda Bhandari, Sanjay Ray Yadav, Krishna Das Manandhar

**Affiliations:** 1 Central Department of Biotechnology, Tribhuvan University, Kirtipur, Kathmandu, Nepal; 2 Department of Molecular Biology and Virology, Centre for Molecular Dynamics Nepal, Thapathali, Kathmandu, Nepal; 3 Department of Tropical and Infectious Disease, Sukraraj Tropical and Infectious Disease Hospital, Teku, Kathmandu, Nepal; 4 Department of Infection and Immunology, Kathmandu Research Institute for Biological Sciences, Lalitpur, Nepal; 5 Department of Haematology and Biochemistry, Chitwan Medical College and Teaching Hospital, Chitwan, Bharatpur, Nepal; Public Health England, UNITED KINGDOM

## Abstract

Dengue virus (DENV) infection is endemic in Nepal. Although infection rates are reported annually, little information is available about the circulating viral serotypes and genotypes. Here, we report the results of a multicentre cross-sectional study of DENV serotypes and genotypes sampled from individuals with suspected DENV infection in Nepal in 2017. Of the 50 patients sampled, 40 were serologically positive for DENV NS1, 29 for anti-DENV IgM, 21 for anti-DENV IgG and 14 were positive by qRT-PCR. The three serotypes DENV-1, 2 and 3 were detected and there was no DENV-4. Positive samples from serotyping were subjected to PCR amplification by envelope (E) gene specific primer and subsequent bidirectional sequencing of 5 samples. A time to most recent common ancestor phylogenetic tree was constructed from the new sequences obtained here together with historical DENV-1 and DENV-2 E gene sequence*s*. The DENV-1 isolates (n = 2) from Nepalese individuals were closely related to Indian genotype V, whereas DENV-2 isolates (n = 3) belonged to Cosmopolitan genotype IVa, which is closely related to Indonesian isolates. Historical DENV isolates obtained between 2004 and 2013 clustered with Cosmopolitan IVb, Cosmopolitan IVa, and Asian II genotypes. All Nepalese isolates had different lineages with distinct ancestries. With the exception of isolates obtained in 2004, all other previously published isolates had ancestry to geographically distant part of the world. Molecular analysis revealed dengue epidemics to be comprised of different genotypes of serotype 1 and 2 raising concerns on potential role of different genotypes causing Dengue hemorrhagic fever. Also, our result indicated spread of DENV-2 in non-endemic area such as hilly region of Nepal which was considered to be free of dengue due to high altitude and cold weather.

## Introduction

Dengue virus (DENV) is transmitted by *Aedes spp*. mosquitoes and causes an estimated 390 million infections annually [1]. The DENV genome is an approximately 11-kb positive-sense, single-stranded RNA molecule [2] that encodes three structural proteins (capsid, membrane, and envelope [E]) and seven non-structural proteins (NS1, NS2A, NS2B, NS3, NS4A, NS4B, and NS5). Among the structural proteins, the E protein plays critical roles in virus attachment and fusion to the host cell membrane and is also the most immunogenic [3]. DENV exists as four serotypes that are further divided into distinct genotypes with sequence divergence not greater than 6% within the chosen genome region [4]. Current genotypes of DENV based on complete envelope gene sequences [5] is as shown in Table 1.

**Table 1:** DENV serotypes and genotypes [5].

Serotype	Genotype	Distribution
**DENV-1**	I	Southeast Asia, China, East Africa
	II	Thailand
	III	Sylvatic strains from Malaysia
	IV	West Pacific islands, Australia
	V	America, strains from West Africa, and a limited number of strains collected from Asia
**DENV-2**	Asian genotype	
	i) Asian 1	Malaysia, Thailand
	ii) Asian 2	Vietnam, China, Taiwan, Sri Lanka and Philippines
	Cosmopolitan	Australia, East and West Africa, Pacific and Indian ocean islands, Indian subcontinent, Middle East
	American	Latin America, Caribbean, Indian subcontinent and Pacific Islands in the 1950s and 1960s
	Southeast Asian/American	Thailand, Vietnam and strains collected in the Americas
	Sylvatic	West Africa and Southeast Asia
**DENV-3**	I	Indonesia, Malaysia, Philippines, South Pacific islands
	II	Thailand, Vietnam and Bangladesh
	III	Sri Lanka, India, Africa and Samoa
	IV	Puerto Rico, Latin and central America and the 1965 Tahiti strain
**DENV-4**	I	Thailand, Philippines, Sri Lanka, Japan
	II	Indonesia, Malaysia, Tahiti, the Caribbean and America
	III	Thailand
	IV	Sylvatic strains from Malaysia

DENV infection was first detected in Nepal in a Japanese traveler in 2004 [6]; this was reported as serotype 2 and cosmopolitan genotype IVb (referred to as genotype 1 in Takasaki et al.) [7]. The report of DENV infection in Nepal in 2006 indicated that all four serotypes were circulating [8] in nine districts of the lowland Terai region, but sequence information was unavailable at that time. Since then, sporadic DENV outbreaks have been reported annually, with major outbreaks occurring in 3-year cycles with a shift in dominant serotype and genotype: DENV-1 genotype V in 2010 [9], DENV-2 Cosmopolitan IVa and Asian II genotypes in 2013 [10] and DENV-1 of unknown genotype in 2016 [11].

Laboratory methods for the diagnosis of DENV infection include viral culture, detection of viral RNA and proteins, detection of host antibodies, or a combination of these methods. During the acute phase of the infection, DENV can be detected in the blood for 4–5 days; thereafter, serology is the main method of diagnosis [12].

In the present study, we aimed to characterize various aspects of DENV infection in Nepal in 2017 by sampling 50 patients who presented with suspected dengue disease at six hospitals throughout Nepal. Patient symptoms, serostatus and demographic data were collected and correlated with DENV serotypes and genotypes after sequencing of the DENV E protein-coding region. These data shed light on the genotypes of DENV circulating in Nepal in 2017 and add vital information on this region to the world-wide DENV sequence database. This molecular surveillance study, which is the first of its type conducted in Nepal, will be of great help in understanding the origins, genetic diversity, transmission dynamics, and epidemic potential of DENV.

## Methods

### Study sites, sample collection, and ethical declarations

This was a cross-sectional descriptive study of samples collected from 50 patients visiting six hospitals in Nepal from September to November 2017, the peak months of the outbreak in that year. The participating sites were: “Sukraraj Tropical and Infectious Disease Hospital (STIDH)”, Kathmandu; “Chitwan Medical College and Teaching Hospital (CMC)”, Chitwan; “Universal College of Medical Science (UCMS)”, Bhairahawa; and three hospitals in Butwal: “Lumbini Zonal Hospital (LZH)”, “Butwal Hospital (BH)” and “National Path Lab (NPL)”. The study protocol was approved by the Nepal Health Research Council (Reg. no. 378/2016). Written informed consent was obtained from all adult patients or from the parent or guardian of children under 18 years of age.

We enrolled patients presenting with an acute febrile illness of 2–7 days duration with symptoms of dengue like muscle and joints pain, nausea, rashes, etc. as mentioned in Table 2 and as suggested by attending physician. Patients were excluded if they were positive for human immunodeficiency virus, aged <5 years or pregnant. The attending physician made the diagnosis and recorded the patient’s demographic and clinicopathological information.

**Table 2:** Presenting signs and symptoms of patients with suspected dengue.

Symptoms	Number (%)
**Dengue without warning signs**	
Fever	50 (100%)
Muscles and joints aches	31 (62%)
Vomiting	26 (52%)
Rash	23 (46%)
Nausea	23 (46%)
**Dengue with warning signs**	
Abdominal pain or tenderness	10 (20%)
Hemorrhage	5 (10%)
Lethargy	5 (10%)
Petechiae	3 (6%)
Gum bleeding	2 (4%)
Liver enlargement (>2 cm)	2 (4%)
Clinical fluid accumulation	2 (4%)
**Severe dengue**	
Fluid accumulation with respiratory failure	2 (4%)
Organ failure	0
Shock	0
Bleeding	0
**WHO classification**	
Dengue without warning signs	32 (64%)
Dengue with warning signs	16 (32%)
Severe dengue	2 (4%)

Disease classification according to WHO 2009 guidelines. See Methods for details.

### Clinical categorization of disease

Patient symptoms were classified according to the World Health Organization (WHO) 2009 guidelines for dengue disease (Table 2): (i) dengue without warning signs (fever with two of the following: nausea, vomiting, rashes, muscle and joint pain), (ii) dengue with warning signs (abdominal pain or tenderness, persistent vomiting, clinical fluid accumulation, mucosal bleeding, lethargy, or liver enlargement >2cm) and (iii) severe dengue (severe bleeding, shock, organ failure, or fluid accumulation with respiratory distress) [12].

### Serological assays and designation of infection status

Anti-DENV IgG and IgM antibodies and DENV NS1 antigen were detected in duplicate samples using standard InBios ELISA kits (Cat. no. DDGS-R, DDMS-1, and DNS1-R, respectively; InBios International, Seattle, WA), according to the manufacturer’s instructions. Immune status ratio (ISR) was calculated according to the kit instructions to classify samples as DENV positive, negative, or equivocal. Primary and secondary infection were defined as ratios of anti-DENV IgM to IgG of ≥1.2 and <1.2, respectively. The ratio was valid for the sera samples diluted in the ratio 1:100 as described by Falconar et al. [13]. Patients who did not show any detectable levels of serum dengue-specific IgM and IgG were classified as seronegative.

### RNA isolation and cDNA synthesis

Viral RNA was extracted from 140 μL of serum using a QIAamp^®^ Viral RNA Mini kit and spin protocol (Cat. No. 52904). Real Time RT-PCR assay (Multiplex) was performed for detection of DENV 1–4 serotypes. For conventional PCR, cDNA was prepared using ProtoScript^®^ First Strand cDNA Synthesis Kit (Cat. No. E6300S) according to the manufacturer’s instructions.

### DENV serotype analysis by multiplex quantitative reverse-transcription polymerase chain reaction

Dengue diagnosis kit (Cat No. KK0128) provided by Center for Disease Control and Prevention (CDC), USA was used for identification of DENV serotypes alongside with positive controls for dengue (DENV-1 to 4) provided with the kit. PCR master mix with final volume of 20 μL per well were prepared [RNase-free water-3.7μL, 2Xs Buffer-12.5μL, Primers Forward and Reverse for DENV-1 to 4 (D1 F/R–D4 F/R)-100 μM, Probe for DENV-1 to 4 (P1-P4) -10 μM, Superscript III Platinum one-step qRT-PCR system enzyme (Invitrogen Cat No. 11732–020)-0.5 μL] in two separate labelled Eppendorf tubes for DENV reaction and Human Specimen Control (HSC) reaction. PCR plate was loaded to Real-Time PCR machine (BIORAD-CFX96 Touch^™^ Real-Time PCR Detection System) having program set of reverse transcription at 50°C for 15 min, inactivation at 95 °C for 2 min followed by 45 PCR amplification cycles (melting at 95°C for 15 sec with ramp rate 4.4°C/s, and annealing and extension temperature at 60°C for 1 min with ramp rate 2.2°C/s); cooling program set at 50°C for 20 min with ramp rate 2.2°C/s) and finally reaction was left at 4°C. The results were interpreted as positive to DENV-1, 2, 3 and 4 if amplification of probes; FAM (Blue), VIC (Green), Texas Red (Red) and Cy5 (Purple) curve were amplified respectively within Cq value 37.

### Primer design and nested RT-PCR

Primers were designed to amplify partial envelope gene of dengue virus. The sequences of representative genotype within different serotypes were prepared from NCBI Genbank. Those sequences were then aligned with Mafft (Multiple Alignment using Fast Fourier Transform) [14] followed by visualization with Aliview v 1.23 [15] and conserved regions were targeted to select primers. The primers were chosen so as to perform nested PCR. Binding position for the primers on the consensus sequence and sequence of the primers along with size of amplified product is shown in supplementary S1 Table. Quality control of primers were performed by using various tools like Primer BLAST [16] and Oligoanalyzer [17]. Nested PCR of the cDNA was performed for all the Real Time RT-PCR positive samples using the envelope protein primers (S1 Table) and Solis Biodyne -5xFIREPol^®^ Master Mix (Cat. No. 04-11-00125). The first round of PCR condition was set to initial denaturation at 95°C for 3 min followed by 35 cycles of the PCR with cycling conditions of denaturation 95°C for 30 sec, annealing 60°C for 45 sec and extension 72°C for 2 min. The final extension was done at 72°C for 10 min and the reaction was kept at hold at 4°C. PCR product was then taken as template for second round PCR and PCR condition was set same except for extension that was done for 1 min. Agarose gel electrophoresis was run in 1.5% agarose and the bands were visualized under UV transilluminator.

### Sequencing and phylogenetic genotyping

Among the PCR positive samples, 5 randomly selected PCR products (2 from DENV-1 and 3 from DENV-2) were further sent to Nepal Academy of Science and Technology (NAST), Nepal for bidirectional sequencing where PCR products were purified and sequenced using the same primers as those used in the PCR performed. DNA sequences were then processed by using software FinchTV v1.4.0 & Bioedit v7.0.5.3 and were visualized in Aliview v1.23 [15]. Genotyping of the sequences were performed by phylogeny method against dengue virus genotyping database [18]. There were 110 and 149 sequences for DENV-1 and DENV-2 respectively which were tallied to get the phylogeny tree (S1 & S2 Figs). DNA sequences of the envelope protein obtained in this study were deposited in GenBank with accession numbers MN507633- MN507637 (S3 Table).

Phylogenetic analyses were carried out and phylogenetic tree was constructed using time to a most recent common ancestor (TMRCA) with an average clock rate of 10^-3^under GTR+I+T93 substitution model [19]. A Bayesian skyline coalescent distribution was implied. Markov Chain Monte Carlo (MCMC) were run for 40 million generation to get adequate RSS (Random Sample Size) value of >100 in BEAST v2.5.1. Separate phylogenetic tree was constructed with tip dates for DENV-1 and DENV-2 against dengue genotype database [18] along with previously reported sequences from Nepal. The runs were visualized for quality by Tracer v1.7.1 and summarized with 25% burnin and node selection were done with greater than 0.44 posterior probability value using Tree annotator v2.5.1. All trees were visualized and edited in Figtree v1.4.3 [20].

### Statistical analysis

Statistical analyses were performed using Prism 7.0 software (GraphPad). Descriptive statistics are reported as the mean ± standard deviation and categorical variables are reported as frequency and percentages.

## Results

### Patient characteristics

**Fig 1:**
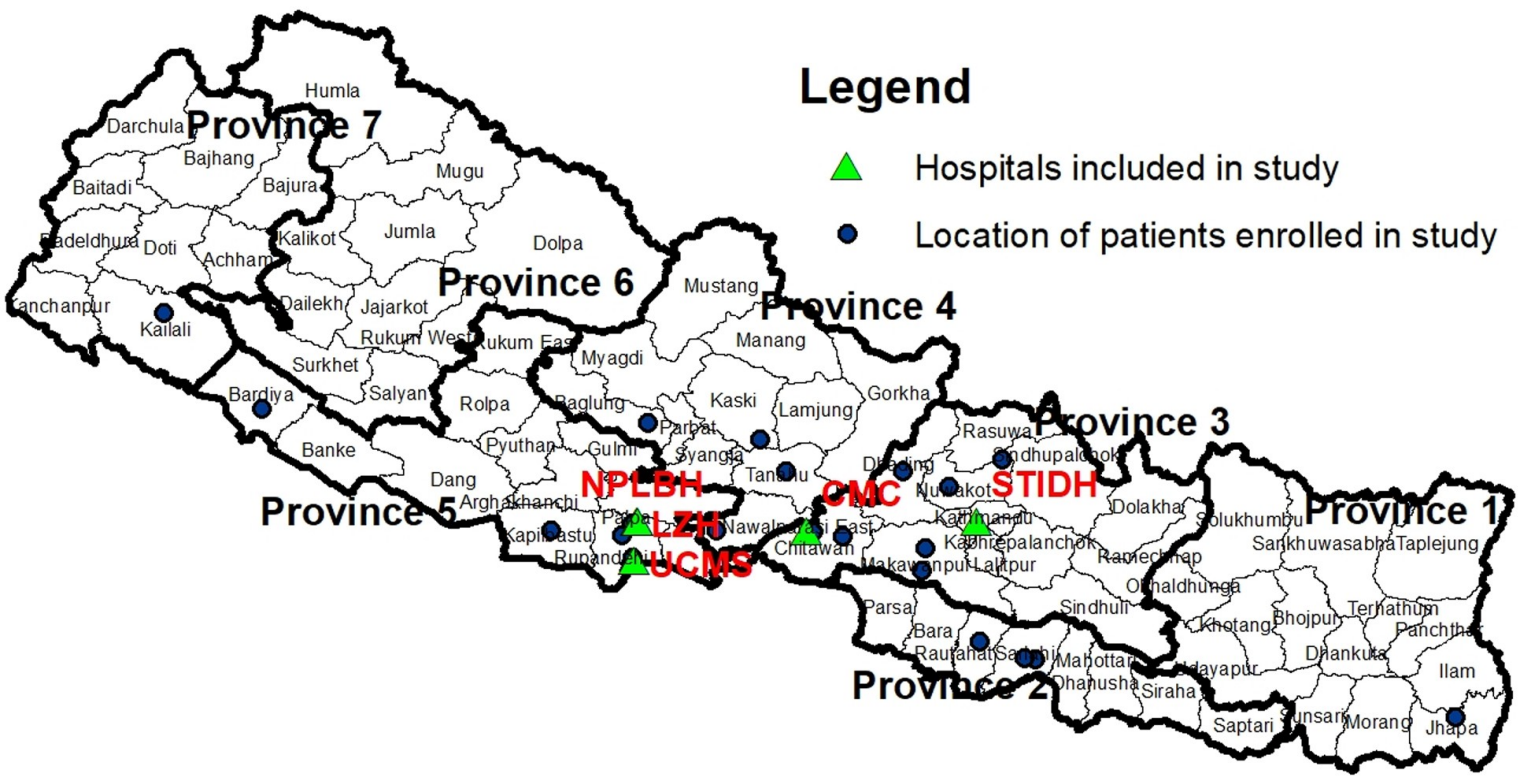
Distribution of the participating sites. Nepal consists of three broad terrains: the southern Terai districts, the central hill districts, and the northern mountain districts. The legends denote the hospitals where samples were collected and the districts where people got infected. The hospitals were: “Sukraraj Tropical and Infectious Disease Hospital (STIDH)”, Kathmandu; “Chitwan Medical College and Teaching Hospital (CMC)”, Chitwan; “Universal College of Medical Science (UCMS)”, Bhairahawa; and three hospitals in Butwal: “Lumbini Zonal Hospital (LZH)”, “Butwal Hospital (BH)” and “National Path Lab (NPL)”. Prepared using Arcmap 10.3 [21] with spatial data obtained from the Government of Nepal official website http://drm.moha.gov.np/ [22].

**Fig 2:**
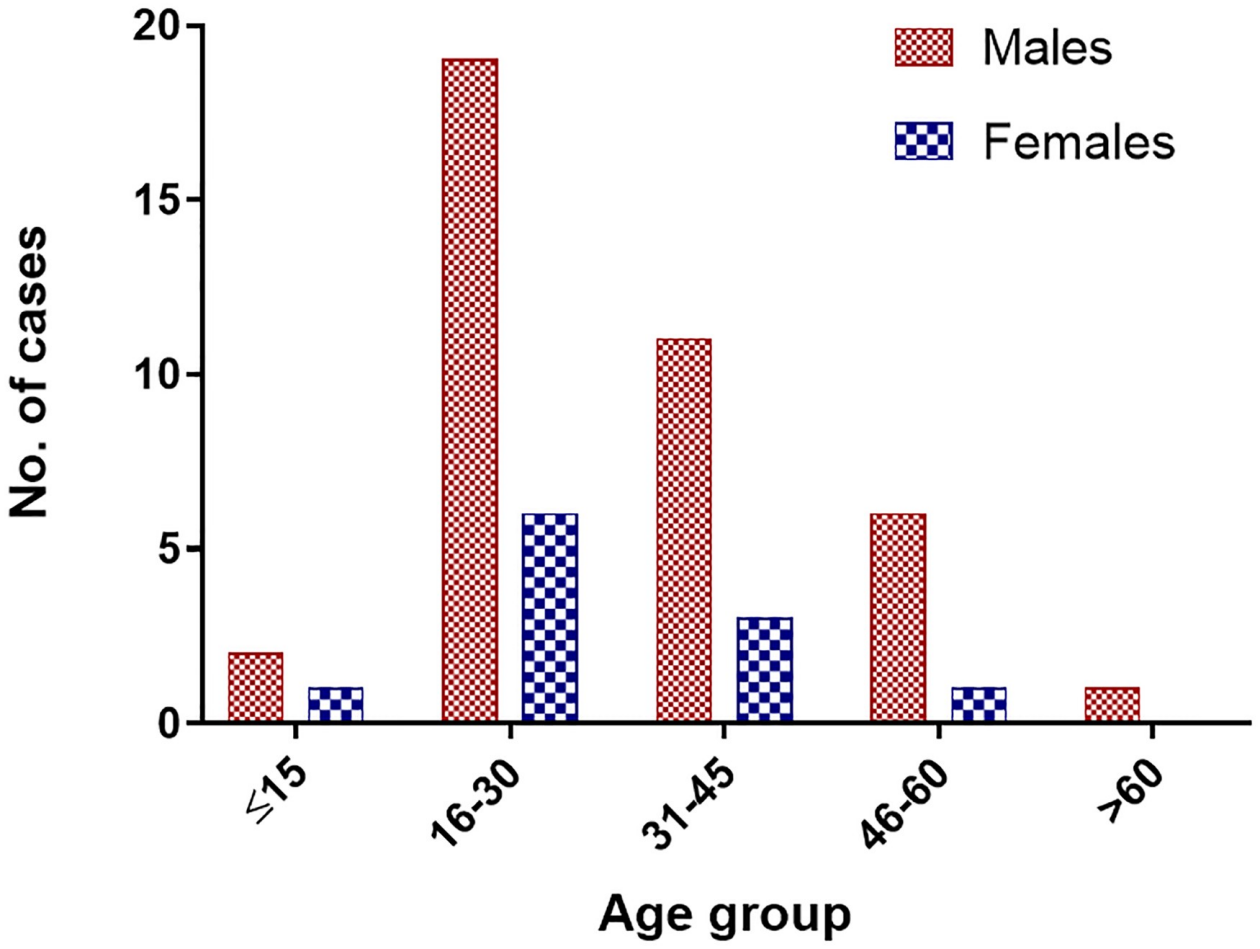
Age distribution of the patients (n = 50).

This hospital-based study enrolled 50 patients with suspected DENV infection seen at six hospitals in Nepal: STIDH (n = 27); CMC (n = 14); UCMS (n = 2); and the three hospitals from Butwal (n = 7). The sampling sites encompass the southern lowland Terai region from East to West and the hilly region in the central zone (Fig 1). The ratio of males to females was 3.5:1 (males 39/50, 78%) and the ages ranged from 12 to 74 years (31.18 ± 13.61 years). The majority of patients of both sexes were age 16 to 30 years (n = 25) followed by 31 to 45 years (n = 14; Fig 2).

### Clinical manifestations of suspected DENV infection

The presenting symptoms of the 50 patients with suspected dengue are shown in Table 2. Other than fever, the most common symptoms were muscle and joint aches (62%), vomiting (52%), nausea (46%), and rashes (46%). Based on these symptoms, 32 (64%), 16 (32%), and 2 (4%) patients were classified as having dengue without warning signs, dengue with warning signs, and severe dengue, respectively.

### IgM, IgG and dengue NS1 antigen serology

**Fig 3:**
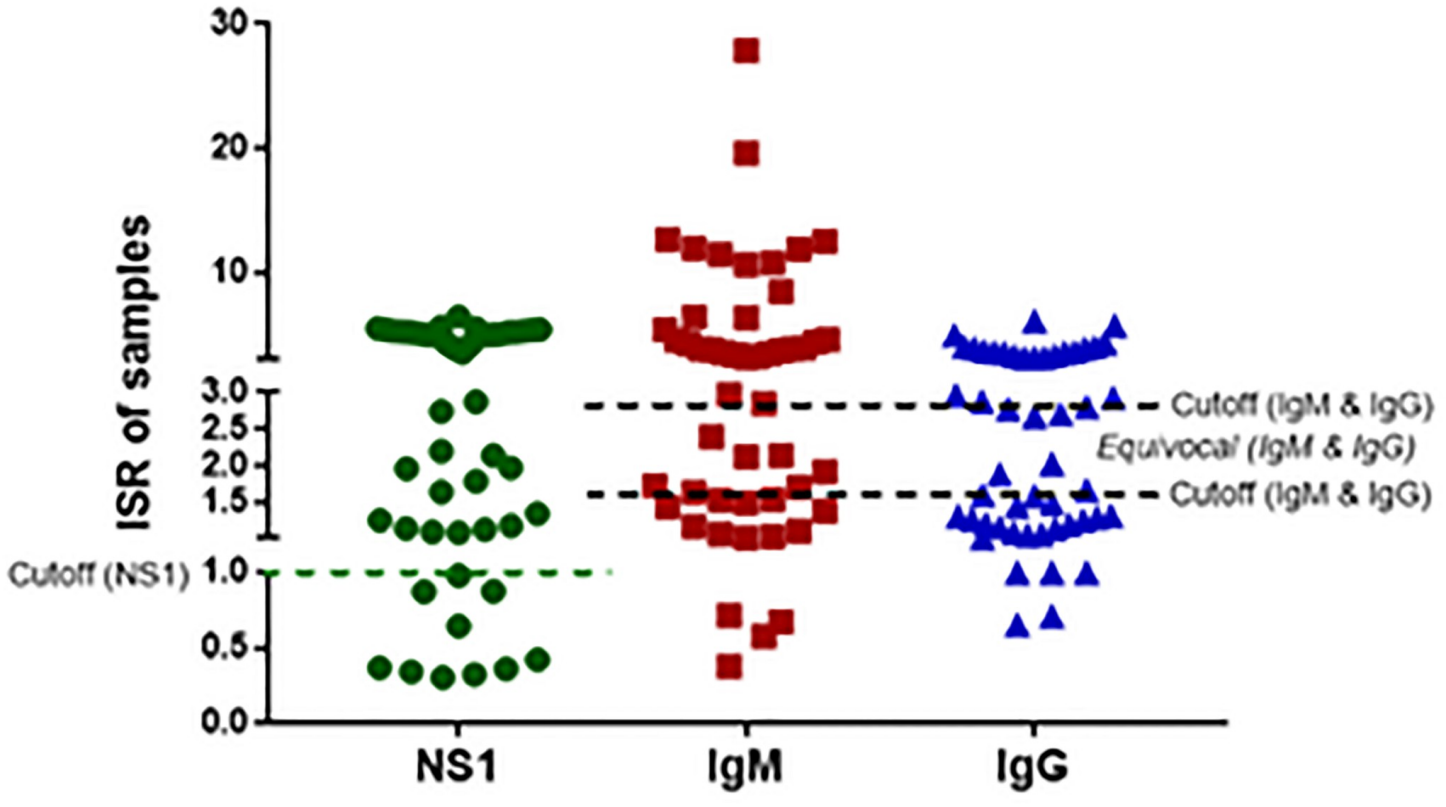
NS1 antigen, anti-DENV IgM, and anti-DENV IgG serostatus of the 50 patients with suspected dengue. The dotted lines represent the cut-off values for positive and negative status for NS1 or positive, negative, and equivocal status for IgM and IgG. ISR, immune status ratio.

Of the 50 patients with suspected dengue, 40 (80%) were positive for NS1. In case of IgM, 29 (58%) were positive, 15 (30%) negative and 6 (12%) were equivocal. For IgG, 21 (42%) were positive, 22 (44%) negative and 7 (14%) were equivocal (Fig 3).

### Frequency of DENV serotypes detected by qRT-PCR and nested PCR

Despite the high frequency of NS1-positive and DENV-seropositive patients, DENV was detected in samples from only 14 of the 50 patients (28%) by qRT-PCR analysis of the DENV performed using the CDC serotyping kit. Of the 14 PCR positive samples, 2 were positive for DENV-1, 11 for DENV-2 and 1 for DENV-3 (Table 3). DENV-4 was not detected in any sample. Serotype-specific nested PCR performed on the samples determined to be DENV positive by qRT-PCR showed amplicons of sizes 546 bp for DENV-1, 927 bp for DENV-2, and 991 bp for DENV-3. None of the patients was concomitantly infected with more than one DENV serotype.

**Table 3:** Frequency and distribution of DENV serotypes detected by qRT-PCR and nested PCR.

Serotype	Districts	Province	Number (%) of patients (n = 50)
DENV-1	Rupandehi	5	2 (4%)
DENV-2	Sarlahi, Chitwan, Jhapa, Makwanpur, Kaski, Dhading, Kapilvastu	1, 2, 3, 4, 5	11 (22%)
DENV-3	Bardiya	5	1 (2%)
DENV -4	-	-	0 (0%)

See Fig 1 for Province locations.

Whereas DENV-1 was detected only in 2 patients from Rupandehi district and DENV-3 was detected only in 1 patient from Bardiya district; DENV-2 had a much broader geographic distribution and was detected in patients from Sarlahi, Chitwan, Jhapa, Makwanpur, Kaski, Dhading, and Kapilvastu. Heterospecific DENV circulation was found in Province 5 (Table 4). DENV-2, which has been the most dominant serotype in Nepal since 2004, was prevalent in the hilly districts of Province 3 (Makawanpur, n = 1 and Dhading, n = 1) and Province 4 (Kaski, n = 1).

**Table 4:** Distribution and infection status of patients with suspected dengue infection.

Districts	Province	Elevation above sea level (m)	Number (%) of cases	NS1 +ve	IgM +ve	IgG +ve	Infection status		
							1°	2°	Neg
Sarlahi	2	<300–1000	11 (22%)	8	4	7	1	8	2
Rupandehi	5	100–1229	9 (18%)	7	7	3	7	1	1
Chitwan	3	<300–2000	7 (14%)	6	4	1	4	1	2
Dhading*	3	488–7809	6 (12%)	6	4	2	3	2	1
Makawanpur*	3	<300–3000	4 (8%)	3	3	3	2	2	0
Nuwakot*	3	300–5000	2 (4%)	0	2	1	2	0	0
Kapilvastu	5	<300–2000	2 (4%)	1	1	1	1	1	0
Jhapa	1	<300–1000	2 (4%)	2	1	0	1	1	0
Kaski*	4	300–6400	2 (4%)	2	1	1	1	1	0
Nawalpur#	4	<300–2000	1 (2%)	1	1	0	1	0	0
Kailali	7	179–1957	1 (2%)	1	0	0	0	0	1
Rautahat	2	<300–1000	1 (2%)	1	0	1	0	1	0
Tanahun*	4	<300–2000	1 (2%)	1	1	0	1	0	0
Bardiya	5	<300–2000	1 (2%)	1	0	0	1	0	0
		Total	50 (100%)	40	29	20	25	18	7

Province refers to regions in Fig 1.

*, hilly districts;

^#^, previously known as Nawalparsi;

1°, primary infection; 2°, secondary infection; Neg, seronegative.

### Regional distribution of patients with primary and secondary DENV infections

The residence and serostatus of the 50 patients are shown in Table 4. DENV was detected in patients from 6 of the 7 provinces of Nepal (exception, Province 6). Of note, although DENV was previously restricted to the plain Terai regions of Nepal, it has spread rapidly; indeed, our data confirm that DENV is now circulating in provinces in the colder hill regions (elevation 100–7809 m). The highest number of DENV-positive patients were from Provinces 2 (n = 12, 24%), 3 (n = 19, 38%), and 5(n = 12, 24%), which encompass the central and southern Terai regions and the southern hill and mountainous regions (Table 3 and Fig 1). The serostatus of patients in most districts (except Sarlahi and Rautahat) was consistent with primary DENV infection (25, 50%), while fewer had secondary infections (18, 36%) or were seronegative (7, 14%). These results support the spread of DENV into the hilly regions of Nepal. While several regions were represented by only one patient with primary infection, the Rautahat district of Terai was the only region in which secondary but not primary DENV infection was detected.

### Phylogenetic and genotypic analysis of DENV serotypes isolated from Nepalese patients

**Fig 4:**
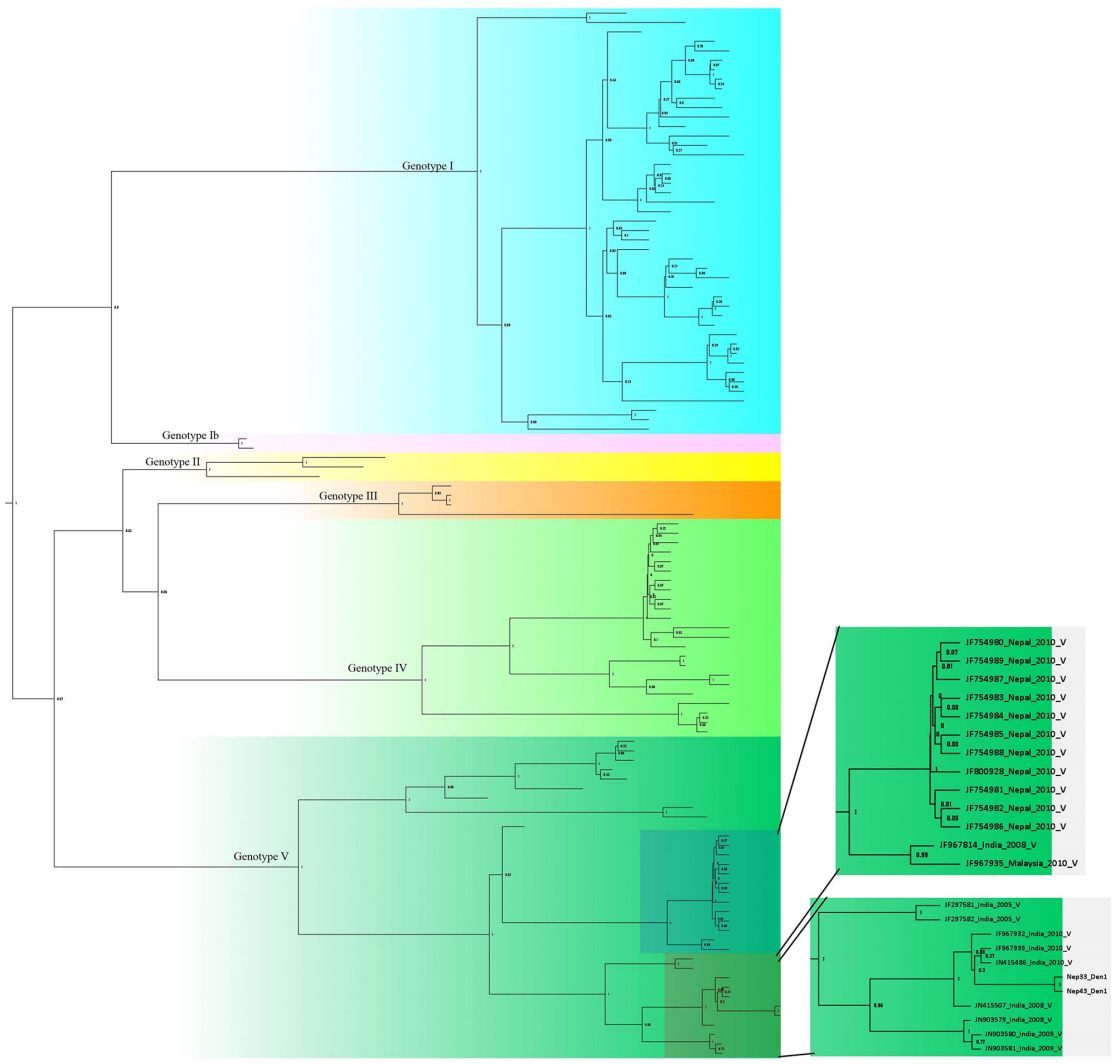
Phylogenetic tree of DENV-1 isolates. The two sections to the right show enlarged images of the lower left boxes containing the Nepal sequences from the present study. Sequences are labeled by accession number, country, year of isolation, and genotype. Tree is labelled with posterior probability at node. Detail tree with all of the sequences used for genotyping is shown in S1 Fig. Constructed using BEAST v2.5.1 with Gamma+I+T93 substitution modeled against the DENV genotyping database.

**Fig 5:**
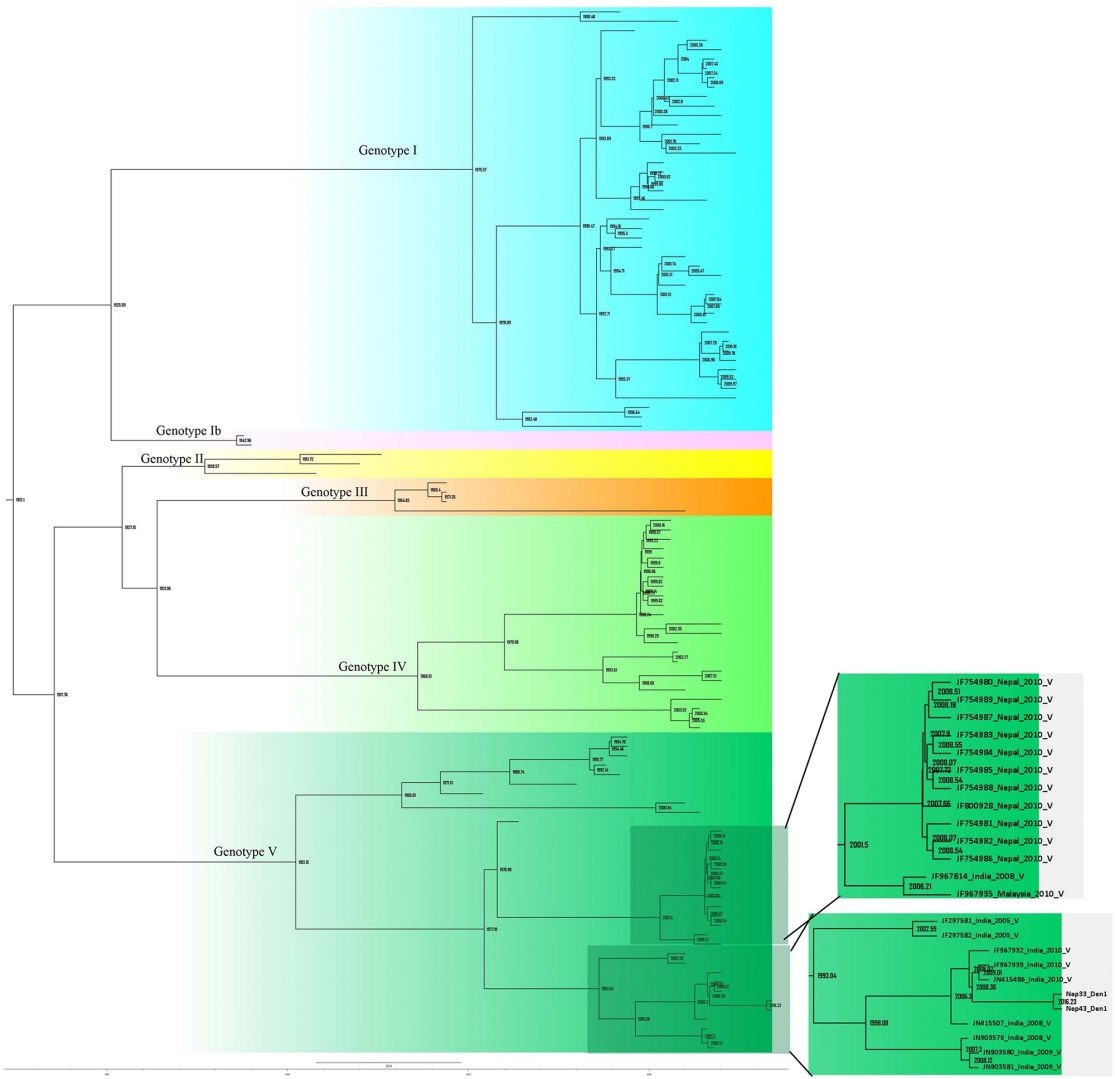
Phylogeny tree of DENV-1 serotype constructed using BEAST v2.5.1 using Gamma+I+T93 substitution model against Dengue genotyping database. Tree is labelled with node age at corresponding node. Detail tree with all of the sequences used for genotyping is shown in S1 Fig.

Bidirectional sequencing and construction of a phylogenetic tree for two DENV-1 isolates showed distinct clades belonging to different genotypes (S1 Fig). DENV-1 reported in this study (Nep_33/Nep_43_Den1) was closely related to the Indian genotype V isolates (JF415486_ India_2010_ V and JF967939_ India_2010_ V) which diverged in approximately 2008 (Figs 4 and 5). DENV-1 genotype V has historically been the most common strain in the Indian subcontinent. Our finding and a previous study indicate that genotype V first appeared in approximately 1950 [23]. DENV-1 isolated from Nepal in 2010 (not this study) also appears to have Indian origin (Closely related to JF967814_India_2008) with common ancestor that split at around 2001.

**Fig 6:**
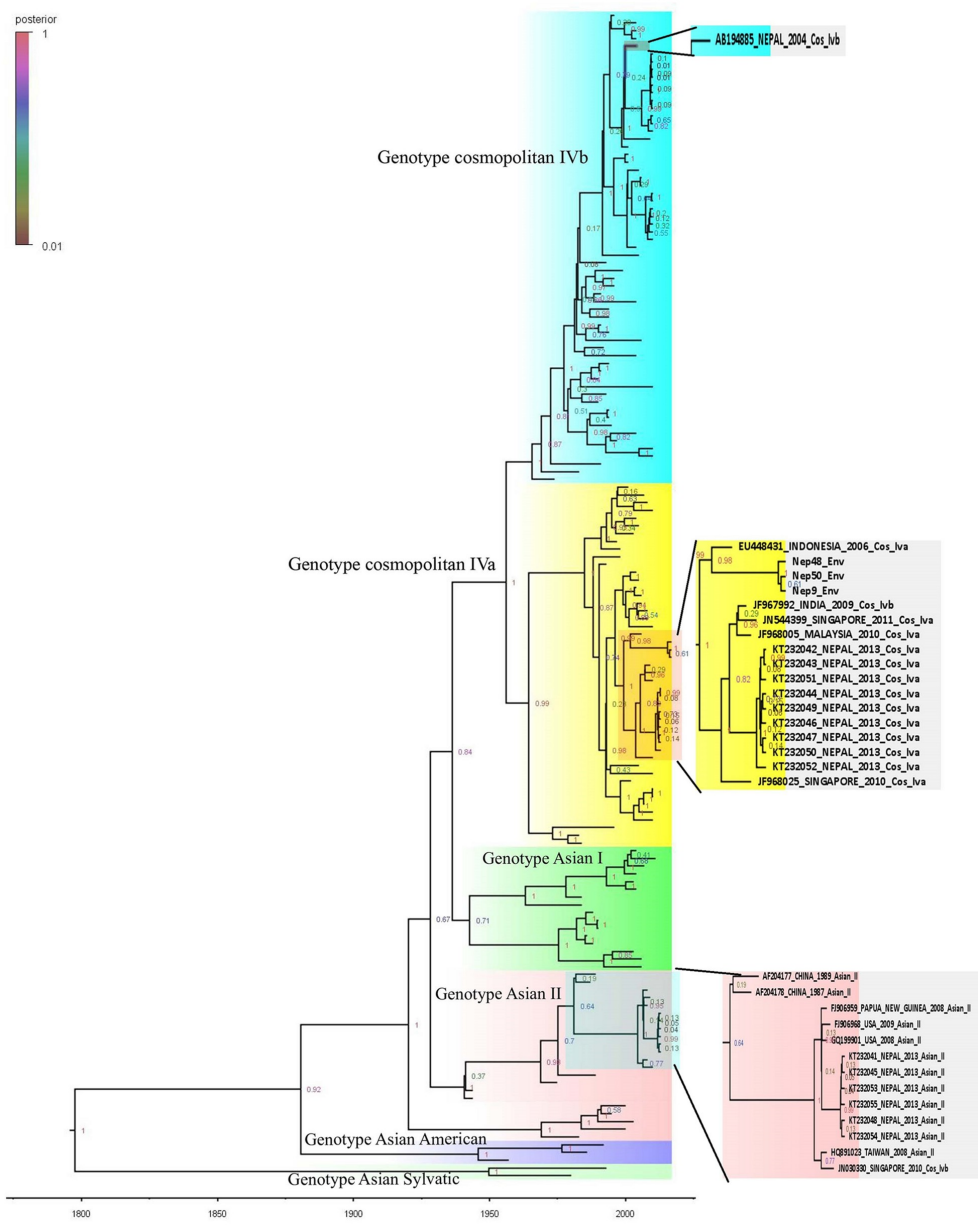
Phylogeny tree of DENV-2 serotype constructed using BEAST v2.5.1 using Gamma+I+T93 substitution model against Dengue genotyping database. All the sequences in the tree are labelled as; accession no._country year of isolation genotype. Tree is labelled with posterior probability at node. Highlighted area in the tree is being zoomed out at right side of figure where sequences from Nepal are shown. Detail tree with all of the sequences used for genotyping is shown in S2 Fig.

**Fig 7:**
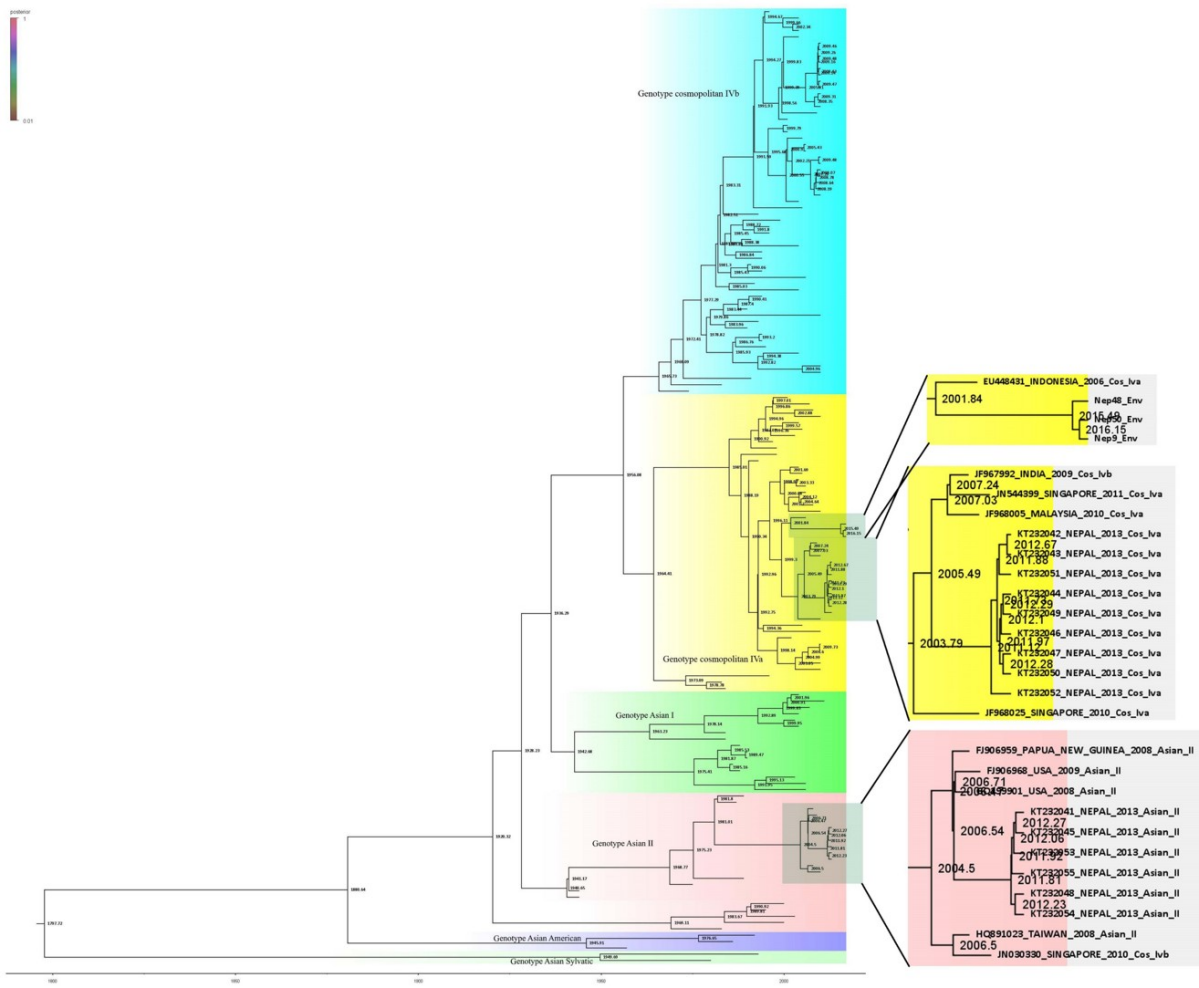
Phylogeny tree of DENV-2 serotype constructed using BEAST v2.5.1 using Gamma+I+T93 substitution model against Dengue genotyping database. Tree are labelled with node age at corresponding node. Detail tree with all of the sequences used for genotyping is shown in S2 Fig.

Three DENV-2 isolates detected and sequenced in this study were more similar to Indonesian isolates of genotype cosmopolitan Iva (Figs 6 and 7). Further, the isolates grouped in different clade than of the Nepal isolate of 2013 cosmopolitan genotype IVa. The 2013 isolate appears to have first diversified at around 2003 whereas 2017 isolates (this study) though belongs to same genotype but clustered in different clade. Further, the other clade of DENV-2 from 2013 belongs to different genotype i.e, Asian II which is closely related to USA and Papua New Guinea Few isolates from India that have also clustered in the same genotype, closely related to 2013 isolates. Moreover, DENV-2 circulating in Nepal appears to be of cosmopolitan IVa, while dominant genotype in Indian subcontinent is cosmopolitan IVb. But, one isolate of DENV-2 is however of genotype cosmopolitan IVb reported in 2004 distantly related to isolates from Singapore and India. In summary, cosmopolitan IVa seems to be predominant genotype of DENV-2 in Nepal with very diverse lineage that is not related to previous isolates.

## Discussion

Many vector-borne infectious diseases circulate in Nepal, a country with one of the lowest GDPs in the world [24]. Dengue was first reported in Nepal in 2004 and has since spread throughout the country [8]. In 2016, DENV infections were reported in 32 districts from the east to the west regions, even into the temperate hill regions [11]. In the present study, we analyzed 50 patients who presented with dengue from September to November 2017 at hospitals in 14 districts representing all provinces except Province6. Despite the small sample number, our findings reflect the rapid expansion of DENV, including the highland hill regions neighboring Kathmandu. The rapid spread of DENV may be due to increased urbanization, better transportation, and increased adaptation of *Aedes* spp. Mosquitoes into these comparatively cold environments.

Consistent with our previous report [25], 78% of our study population was male. Possible explanations include the more likely infection of outdoor field workers, who tend to be male, and the better and quicker access to healthcare afforded to males compared with females. The most frequently infected age group in our study was the 16 to 45 years-old, which is also the most economically productive age group. This highlights the potentially crippling effect of DENV infection on the economy of Nepal through lost productivity [26]. In contrast to the greater prevalence of secondary infections compared with primary DENV infections recorded in earlier years [26], our study of 2017 indicated that more than 50% (25 of 43 seropositive patients) presented with primary DENV infection. This finding might suggest a rise in new infections and even signal the potential for large outbreaks in the near future. Authorities should be alert to this possibility and take precautions to be ready for the next cyclic outbreaks, based on the 2010, 2013 and 2016 outbreaks [26].

The dominant serotypes in the years 2010, 2013, and 2016 outbreaks were DENV-1, DENV-2 and DENV-1 respectively. Interestingly, we detected DENV-1, 2 and 3serotypes in our sample in 2017. Although the DENV serotypes circulating in Nepal have been reported only for 2006, 2010, 2013, and 2016, however, this is second reporting of DENV-3 since 2006 [8].

In contrary to DENV serotypes, little is known about the genotypes and clades of DENV circulating in Nepal. Previous studies reported the presence of DENV-2 Cosmopolitan IVb genotype in 2004 (genotype 1 in Takasaki et al. [7]), DENV-1 genotype V in 2010 [9], and Cosmopolitan IVa and Asian II genotypes in 2013 [10]. In the present study, (Nep 33/43_2017), DENV-1 groups in genotype V, which grouped in different clade than the DENV-1 of Nepal seen in the year 2010 [9]. DENV-1 reported from Nepal in 2010 is closely related to Indian isolate reported in 2008 (JF967814_India_2008) along with Malaysian isolate (JF967935_Malaysia_2010_V) reported in 2010 itself. Nepal isolate of DENV-1 and above two isolates (Indian and Malaysian) had common ancestor at 2001. DENV-1 reported in 2010 and this study appears to be introduced in Nepal independently as they appear to have common ancestor at approximately 1976 (Figs 4 and 5). Furthermore, given history of Dengue epidemics, it is well known that Dengue distribution and dominant serotype as well as genotype has been shifting with time [27].

Given geographic proximity to India, it is expected to have DENV isolated being reported in Nepal having ancestral origin in India. However, DENV-2 reported in this study (Nep_9/48/50_Env) do not have ancestral origin in India. Furthermore, DENV-2 reported in this study belonged to genotype IVa having ancestral origin with Indonesian and other East Asian strains. DENV-2 detected in this study and reported in 2013 epidemic [28,10] is not from same lineage having common ancestor around the year 1999. First report of Cosmopolitan genotype IVa in Nepal was in 2013 while earlier in 2004 genotype IVb strains were reported. Asian II genotype of DENV-2 was also reported in 2013 which was similar to USA/Papua New Guinea strain and the detection in Nepalese territory could be due to accidental spill over probably by tourists. As in case of DENV-1, DENV-2 lineages also show nearest common ancestor before 2004 indicating co-circulation of different dengue strains. The diverse nature of isolates observed might be due to multiple entry of pathogen from different sources because of globalization and ever-increasing dynamic movements of people. These type of diversity in dengue virus holds risk of sudden resurgence of severe dengue disease in near future due to antibody-dependent enhancement (ADE) [29,30]. Particularly, DENV-2 which showed three different genotypes reported so far, with diverse lineage within genotype could be of greater concerns.

One aim of this study was to determine the prevalence of DENV serotypes/genotypes in the temperate highland hill regions of Nepal. We found that DENV-1 and DENV-3 were circulating in the lower belts of Terai, whereas DENV-2 had spread to hill districts such as Dhading. DENV-2 was genotype cosmopolitan IVa, which, unlike Cosmopolitan IVb found in India, is closely related to genotypes found in Southeast Asia (Malaysia, Singapore), indicating an unusual spread of DENV-2 into Nepal. The spread of DENV to higher altitudes could be at least partly attributed to increases in global temperatures (global increase of 0.94°C till 2016 [31]) and local temperatures, possibly due to the increase in population density surrounding the Kathmandu valley. In addition, DENV reported from hilly region all belonging to DENV-2 genotype cosmopolitan IVa might hold answer to why DENV is spreading to previously non-epidemic area. DENV-1 reported from Terai region in this study however agrees with previous reports. Despite the limited number of patients sampled in this study and the genotyping of only DENV-1 and DENV-2 serotypes, our findings identifying specific clades of DENV genotypes in Nepal make an important contribution to our understanding of the current epidemiological status of DENV infection and potential future outbreaks in this region.

## Conclusion

DENV is rapidly spreading in Nepal, especially among the most productive age group, which is a major public health concern. However, there are currently no specific programs to control the epidemic. In this study, we showed that DENV-1, 2 and 3 serotypes were co-circulating in Nepal in late 2017, and we have reported multiple genotypes of DENV-1 and DENV-2. From phylogeny, the DENV-1 isolates from Nepal were closely related to Indian isolates belonging to genotype V whereas DENV-2 detected in this study belonged to genotype cosmopolitan IVa which is closely related to Indonesian isolates. Further, DENV-2 isolates of 2004 and 2013 clustered with genotype cosmopolitan IVb, cosmopolitan IVa and Asian II. All the DENV-2 isolates had different lineages having distinct ancestry from one another. In contrast, except for isolate from 2004, all other had ancestry to geographically distant part of the world. In summary, molecular analysis showed diverse population of DENV surfacing at different time point causing epidemic indicating potential needs to address role of genotype in dengue virus disease dynamics including dengue hemorrhagic fever. The results also show impact of global circulation of virus and its spread in previously non-endemic area, the hilly regions of Nepal. Whole experiments were carried out within Nepal with currently available little information on genetic diversity using coding region of envelope protein. This study helped shed light on picture of dengue infection of 2017. Data generated in this study will help support future viral surveillance and epidemiological investigations of dengue in Nepal.

## Supporting information

S1 Fig Phylogeny tree of DENV-1 serotype constructed using BEAST v2.5.1 using Gamma+I+T93 substitution model against Dengue genotyping database. All the sequences in the tree are labelled as; accession no._country year of isolation genotype. Tree are labelled with posterior probability at node.
(TIF)

S2 Fig Phylogeny tree of DENV-2 serotype constructed using BEAST v2.5.1 using Gamma+I+T93 substitution model against Dengue genotyping database. All the sequences in the tree are labelled as; accession no._country year of isolation genotype. Tree are labelled with posterior probability at node.
(TIF)

S1 Table Primers used for dengue virus confirmation and serotype specific PCR. (DOCX)

S2 Table Cycle threshold (CT) values for Real time PCR positive samples. (DOCX)

S3 Table Details of samples whose sequencing has been performed in this study. (DOCX)
