# Supplementary figures and images for: Molecular phylogeny and distribution of dengue virus serotypes circulating in Nepal in 2017
Source: PLoS One. 2020 Jul 7;15(7):e0234929. doi: 10.1371/journal.pone.0234929 (PMC7340289; doi:10.1371/journal.pone.0234929)

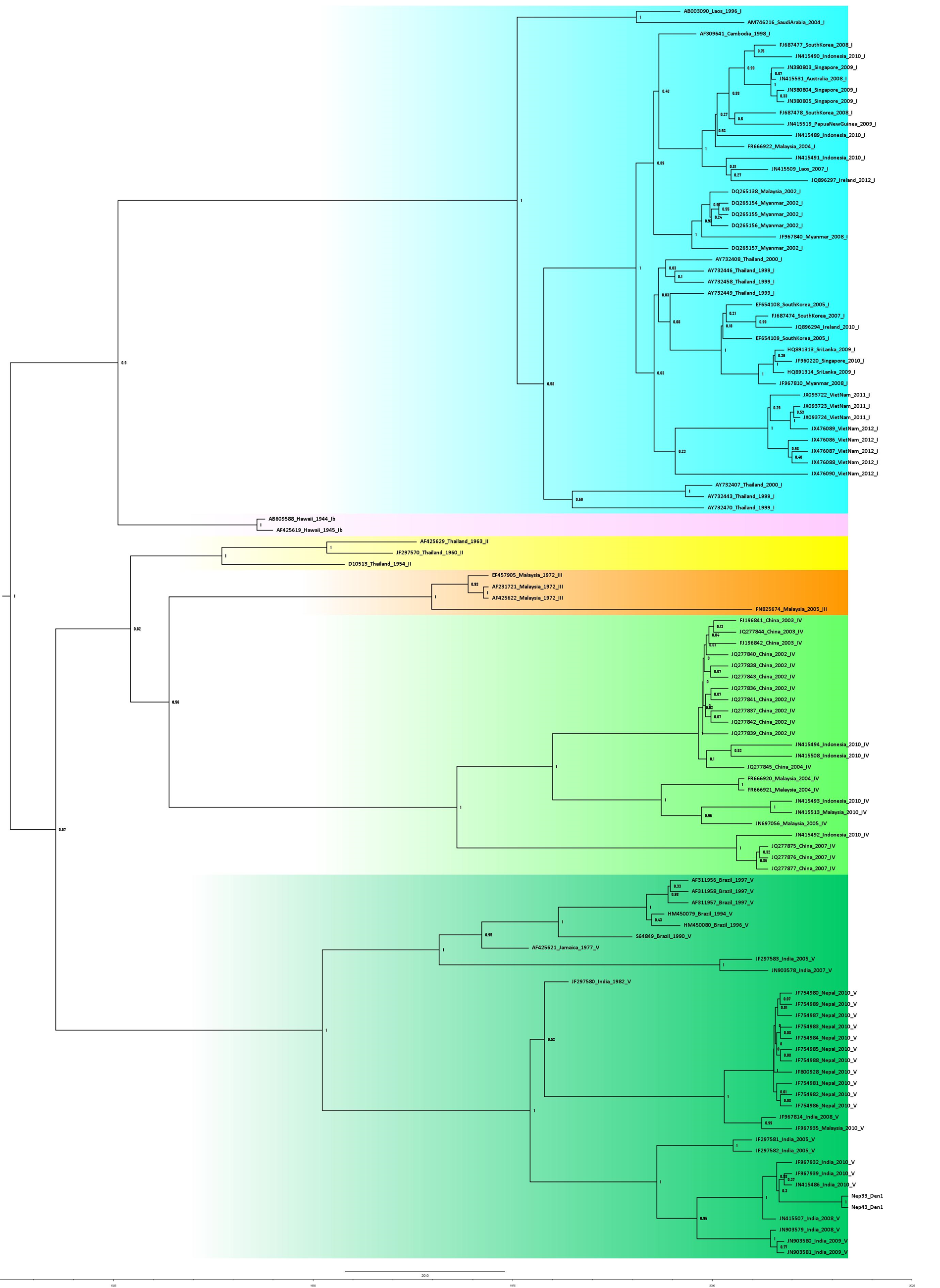

Supplement: S1 Fig — All the sequences in the tree are labelled as; accession no._country year of isolation genotype. Tree are labelled with posterior probability at node. (TIF) [file pone.0234929.s001.tif]

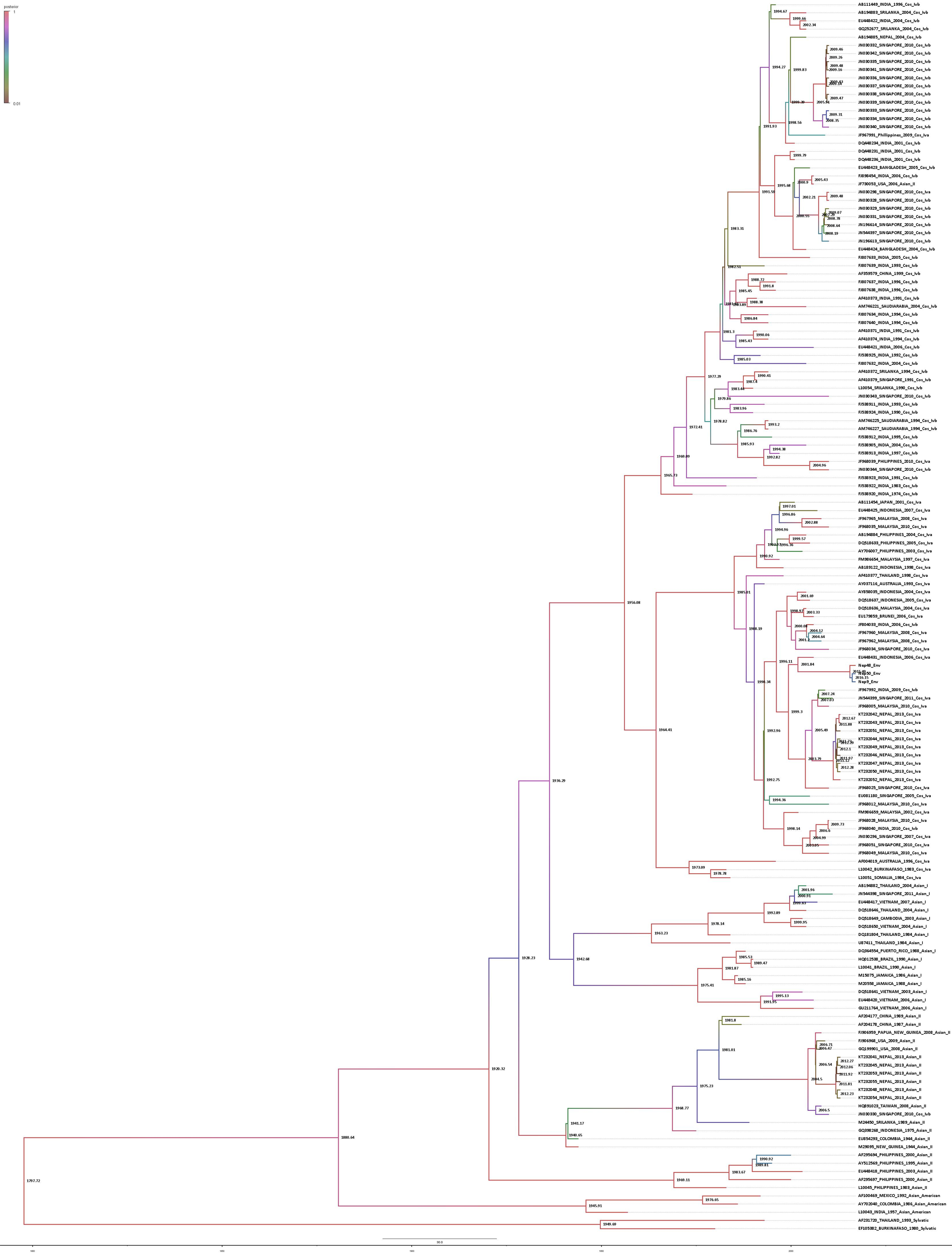

Supplement: S2 Fig — All the sequences in the tree are labelled as; accession no._country year of isolation genotype. Tree are labelled with posterior probability at node. (TIF) [file pone.0234929.s002.tif]
