# Supplementary material for: Molecular phylogeny and distribution of dengue virus serotypes circulating in Nepal in 2017
Source: PLoS One. 2020 Jul 7;15(7):e0234929. doi: 10.1371/journal.pone.0234929 (PMC7340289; doi:10.1371/journal.pone.0234929)
Supplement: S1 Table — (DOCX) [file pone.0234929.s003.docx]

**Supplementary Table S 1: Primers used for dengue virus confirmation and serotype specific PCR**

| **Name of Primers** | **Primer Sequence** | **Location(w.r.t. reference sequence)** | **Expected size of the amplicon (bp)** |
| --- | --- | --- | --- |
| DENV1_RN_F1 | ACAC CAT CCA TGG CCA TGC G | 922-941 | 1501 |
| DENV1_RN_R1 | C CGC CTG AAC CAT GAC TCC | 2401-2421 |  |
| DENV1_RN_F2 | GCG CAA ACT GTG CAT TGA AGC | 1104-1124 | 546 |
| DENV1_RN_R2 | AC CAG CAA ATC TTG TCT GTT CCA | 1627-1649 |  |
| DENV2_RN_F1 | CGCTCCTTCAATGACAATGCG | 1242-1263 | 1523 |
| DENV2_RN_R1 | A ACT CAC AAC GCA ACC ACT ATC | 2152-2172 |  |
| DENV2_RN_F2 | CAG AGG ATG GGG AAA TGG ATG | 936-958 | 927 |
| DENV2_RN_R2 | CT CAT TGT TGT CTC GAA CAT TTG | 2438-2458 |  |
| DENV3_RN_F1 | TGGACATAGAGCTCCAGAAGAC | 1055-1075 | 1332 |
| DENV3_RN_ R1 | G TGT GAT GAT TCC TAT CAC AAT GCA | 2364-2387 |  |
| DENV3_RN_F2 | CTG AGG AGC AGG ACC AGA AC | 1182-1201 | 991 |
| DENV3_RN_ R2 | AA GAT GGC CAT GCG CCT TGC | 2153-2172 |  |
| DENV4_RN_F1 | TGG TCG CCC CAT CTT ACG G | 920-938 | 1526 |
| DENV4_RN_R1 | TGA CAC CAC ACA ACC CAT GTC | 2433-2453 |  |
| DENV4_RN_F2 | CAA GAT GTC CAA CGC AAG GAG A | 1154-1175 | 537 |
| DENV4_RN_R2 | T CAC ATC CTG TCT CTT GGC ATG | 1675-1696 |  |
