# Supplementary material for: Molecular phylogeny and distribution of dengue virus serotypes circulating in Nepal in 2017
Source: PLoS One. 2020 Jul 7;15(7):e0234929. doi: 10.1371/journal.pone.0234929 (PMC7340289; doi:10.1371/journal.pone.0234929)
Supplement: S2 Table — (DOCX) [file pone.0234929.s004.docx]

**Supplementary Table S 2: Cycle threshold (CT) values for Real time PCR positive samples**

| **Subject ID** | **Fluorophore** | **Serotype** | **CT value** |
| --- | --- | --- | --- |
| Nep-7 | Texas red | DENV3 | 23.33 |
| Nep-9 | VIC | DENV2 | 22.37 |
| Nep-26 | VIC | DENV2 | 21.06 |
| Nep-33 | FAM | DENV1 | 26.23 |
| Nep-39 | VIC | DENV2 | 28.62 |
| Nep-40 | VIC | DENV2 | 31.21 |
| Nep-41 | VIC | DENV2 | 33.68 |
| Nep-43 | FAM | DENV1 | 20 |
| Nep-45 | VIC | DENV2 | 29.73 |
| Nep-46 | VIC | DENV2 | 30.72 |
| Nep-47 | VIC | DENV2 | 30.5 |
| Nep-48 | VIC | DENV2 | 22.2 |
| Nep-49 | VIC | DENV2 | 29.41 |
| Nep-50 | VIC | DENV2 | 18.43 |
