# Supplementary material for: Molecular phylogeny and distribution of dengue virus serotypes circulating in Nepal in 2017
Source: PLoS One. 2020 Jul 7;15(7):e0234929. doi: 10.1371/journal.pone.0234929 (PMC7340289; doi:10.1371/journal.pone.0234929)
Supplement: S3 Table — (DOCX) [file pone.0234929.s005.docx]

**Supplementary Table S3: Details of samples whose sequencing has been performed in this study**

| **Sample ID** | **GenBank accession** | **Serotype** | **Genotype** | **Length** |
| --- | --- | --- | --- | --- |
| Nep 33 | MN507633 | DENV-1 | Genotype V | 516 bp |
| Nep 43 | MN507634 | DENV-1 | Genotype V | 515 bp |
| Nep 48 | MN507635 | DENV-2 | Cosmopilitan genotype IV a | 871 bp |
| Nep 50 | MN507636 | DENV-2 | Cosmopilitan genotype IV a | 879 bp |
| Nep 9 | MN507637 | DENV-2 | Cosmopilitan genotype IV a | 886 bp |
